# Does internet use benefit health?—PSM-DID evidence from China’s CHARLS

**DOI:** 10.1371/journal.pone.0306393

**Published:** 2024-07-09

**Authors:** Yinkai Liao, Nengsheng Luo

**Affiliations:** School of Economics and Trade, Hunan University, Changsha, Hunan, China; Ladoke Akintola University of Technology Teaching Hospital: LAUTECH Teaching Hospital, NIGERIA

## Abstract

Amid the increasing global internet penetration, understanding the impact of internet use on residents’ health is crucial. This aids in formulating more effective health policies and provides empirical evidence for promoting health equity and improving overall public health. Drawing on the China Health and Retirement Longitudinal Study (CHARLS), this paper employs the Propensity Score Matching-Difference in Differences (PSM-DID) method to examine the impact of the internet on individual health and further explores the pathways through which the internet affects health. We introduce the research background and significance in the introduction. Then, in the theoretical analysis, it incorporates internet variables into the Becker health demand model to analyze changes in health demand and impact pathways. The empirical analysis tests the theoretical findings, leading to empirical results. Finally, the study discusses the results and provides relevant recommendations. The findings indicate significant positive effects of the internet on both physical and psychological health. These effects are realized through reducing health information asymmetry, lowering health costs, and increasing exposure to health-promoting environments. In the heterogeneity analysis, economic-related internet content shows a significant positive impact on resident health. Intensive internet use adversely affects psychological health. The beneficial effects of the internet on health are more pronounced among older individuals, those covered by medical insurance, and regions with higher levels of digital economy. Based on these findings, the study offers policy recommendations concerning individuals’ internet use patterns, the digital evolution of the healthcare industry, and government infrastructure development.

## Introduction

Identifying the determinants of health is pivotal for the treatment of both physiological and psychological ailments, as well as for the improvement of the overall health status of the population. The World Health Organization (WHO) reports that global life expectancy at birth has risen from 67 years in 2000 to 73 years in 2019. However, this increase in life expectancy does not obscure the current health challenges faced by many. It has been reported that the number of individuals with hypertension worldwide has surged from 650 million in 1990 to 1.3 billion in 2019. Additionally, the incidence of psychological health disorders has been on the rise over the past few decades. By 2022, over 1 billion people globally had been affected by some form of psychological illness.

While there has been consistent progress in the fields of economy and technology, health concerns remain increasingly prominent. One of the most notable outcomes of economic and technological advancements has been the rapid digitization of information. In 2022, there were 5.16 billion internet users, accounting for 64.4% of the global population, marking a growth of 1.9% from the previous year. The proliferation of the internet has transformed lifestyles, deeply embedding itself into facets of work, consumption, and social interactions. This profound and intricate influence on life inevitably bears significant repercussions on health (House et al., 2002) [1]. The efficiency, accessibility, and resource-integration capabilities of the internet allow the healthcare industry to offer enhanced services for people. Artificial intelligence optimizes both working and living environments, while technological advancements reduce medical costs for individuals. In essence, the internet era brings forth numerous benefits conducive to enhancing health.

However, in a modern context, “health” encompasses a holistic view, extending beyond the traditional definition of merely being free from illness. This contemporary interpretation holds profound temporal significance, emphasizing the importance of maintaining both physiological and psychological well-being. Questions arise concerning the impact of internet involvement on physical and psychological health, the potential pathways of these influences, and how individuals can navigate the digital landscape to maximize benefits and mitigate potential pitfalls. This paper aims to delve deep into these pertinent issues.

In the domain of internet usage and health, there is a growing literature, with most studies focusing on the relationship between internet use and psychological health. Conversely, attention to the physiological health of people remains limited. In terms of psychological health research, Minto (2015) posits that frequent internet usage can decrease anxiety levels in patients with cardiomyopathy [2]. Studies by Tsai (2014) suggest that people who often use the internet are less likely to suffer from depression [3]. Lagoe and Atikin (2015), using a sample of 245 American people and structural equation modeling, investigated the psychological determinants of using online health information [4]. Their findings indicate a significant association between anxiety and internet use but highlight the internet’s potential to alleviate stress and reduce levels of depression and loneliness. In the realm of physical health research, a study by Kontos and Emmons (2010) notes significant health disparities between those who use the internet and those who do not [5]. Dutta-Berman (2004) argues that individuals with strong health awareness who utilize the internet to search for health information can positively influence resident health [6]. Bender (2011) suggests that online social interactions can help alleviate pain [7]. Additionally, Huang (2020) and Li et al. (2022) examined the effectiveness of technology-based interventions during the COVID-19 pandemic [8, 9]. These studies provide insights into the potential benefits and drawbacks of relying on the internet for health information and services.

The impact of the internet on resident health manifests primarily in three ways: First, the internet reduces information asymmetry by breaking the medical community’s monopoly on specialized information, thus bridging the information gap between doctors and patients, aiding people in better managing their health (McMullan, 2006; Castleton and Fong, 2011) [10, 11]. Furthermore, health information obtained from the internet assists individuals in understanding and applying health advice to their daily lives, leading to healthier lifestyles (Chou et al., 2011; Beck et al., 2014) [12, 13]. Second, as a communication tool, the internet can alleviate feelings of loneliness, depression, and anxiety, thereby enhancing users’ overall health (Oh et al., 2013; Mccloud et al., 2016) [14, 15]. Lastly, with the ongoing development of the internet, new applications continually emerge, with certain intelligent health management programs having a tangible effect on health (Xie et al. 2021) [16].

While existing research highlights the positive effects of internet use on health, other studies emphasize the adverse implications stemming from its inappropriate usage. The internet has undeniably facilitated the rapid dissemination of health information, thus promoting health awareness. However, it’s crucial to rigorously evaluate the veracity and source of information online (Korp, 2005) [17]. This has led to increased informational disparities among different user groups, potentially heightening anxiety (Rider et al., 2014) [18]. Furthermore, excessive internet use has been linked to visual impairments (Bener et al., 2010; Kawashima, 2013) [19, 20]. Research led by Do et al. (2013) suggests that prolonged internet use could potentially reduce individual sleep durations, thereby negatively affecting self-assessed health and predisposing individuals to psychological disorders [21]. This trend is especially pronounced in Asian countries such as South Korea and China (Shaw and Black, 2008; Block and J.M.D, 2008) [22, 23]. Additionally, Zheng et al. (2016) found a correlation between internet use and conditions like eye dryness and neck pain [24].

Previous scholars have made significant contributions to the study of the internet and its impact on public health, yet certain shortcomings in the existing research remain evident. First, much of the research on the internet focuses heavily on economic development and income issues, largely overlooking the effects on quality of life and the impact on human capital. Second, most studies on public health concentrate on psychological factors or self-assessment of health, lacking objective data on physiological health conditions. Third, the primary emphasis has been on the informational effects brought about by the internet, neglecting other potential impacts arising from internet usage.

This study, rooted in data collected through the China Health and Retirement Longitudinal Study (CHARLS), employs a methodological approach grounded in propensity score matching difference-in-differences (PSM-DID) to investigate the nexus between Internet use and health. Notably, this research seeks to introduce several innovative elements. Firstly, contemporary research on the Internet and digitization primarily accentuates macroeconomic ramifications, often failing to consider their intricate impacts on the daily lives of people, especially concerning physical health. This research pioneers a perspective shift by scrutinizing the effect of Internet use on health. Unlike existing studies, which mainly focus on macroeconomic effects, this study delves into the micro-level impacts on individual health. Secondly, the utilization of data sourced from the CHARLS survey enables a microscopic examination of individual behaviors, which forms the basis for employing the difference-in-differences methodology to disentangle the effects of Internet usage on individual health. This approach is relatively rare in existing literature, particularly in analyzing the impact of the Internet on health. Thirdly, the study aims to deconstruct the pathways through which residents’ involvement in the digital realm molds their health, thereby elucidating the mechanisms that govern individual decision-making processes. This contrasts with many existing studies that remain at the descriptive statistical level and do not deeply explore specific mechanisms. Lastly, the research aspires to unravel the heterogeneous effects of Internet use, taking into account factors such as the category of online content accessed, the intensity of usage, personal characteristics, and the levels of digital economic development within different regions. Such detailed heterogeneity analysis is relatively uncommon in the existing literature. Given the ongoing process of digitization, this research becomes pivotal in ensuring the welfare of people, underpinning health, and concurrently, it assumes critical importance in bolstering the digitization efforts in healthcare.

The paper proceeds as follows: the theoretical analysis is presented first, followed by a detailed description of the empirical strategy and data. Next, the primary empirical findings are provided, and mechanisms and heterogeneity are further examined. A discussion of the overall study and its limitations follows. The final part concludes with recommendations for platforms and services.

## Theoretical analysis

In this section, we first analyze Becker’s health demand model to discuss the impact of the internet on health demands. We next delve into the theoretical mechanisms underlying the internet’s effect on public health.

### Becker health demand model

Based on the Becker Health Demand Model [25], individual needs are categorized into health-related requirements and other consumer goods needs. Within the constraints of income and time, individuals aim to maximize their personal utility level. The components of the model include the individual utility function, household production function, income constraint, and time constraint, as follows:

$$\begin{array}{c} U=U(H,Z) \end{array}$$ (1)

$$\begin{array}{c} H=G_{1}(M,T_{h};E) \end{array}$$ (2)

$$\begin{array}{c} Z=G_{2}(X,T_{z};E) \end{array}$$ (3)

$$\begin{array}{c} P_{m}M+P_{x}X=R=N+WT_{w} \end{array}$$ (4)

$$\begin{array}{c} T=T_{w}+T_{h}+T_{z} \end{array}$$ (5)

In Eqs (1)–(5), *U* is an individual’s utility level; *H* is health status; *Z* signifies other consumer goods; *M* is the medical service required to produce health; *X* is the goods necessary for producing *Z*; *T*_*h*_ and *T*_*z*_ are the time required for producing health and other consumer goods, respectively; *E* is other exogenous variables; *T*_*w*_ is the work time; *R* is total individual income; *P*_*m*_ and *P*_*x*_ are the prices per unit of medical service *X*, *W* is the wage rate; *N* is non-wage income, and *T* is the total available time for an individual. After using the internet, personal internet time can be expressed as:

$$\begin{array}{c} T_{int}=\varphi_{int}T \end{array}$$ (6)

$$\begin{array}{c} T_{intw}=\varphi_{intw}T_{w} \end{array}$$ (7)

$$\begin{array}{c} T_{inth}=\varphi_{inth}T_{h} \end{array}$$ (8)

$$\begin{array}{c} T_{intz}=\varphi_{intz}T_{z} \end{array}$$ (9)

$$\begin{array}{c} T_{int}=T_{intw}+T_{inth}+T_{intz}=\varphi_{intw}T_{w}+\varphi_{inth}T_{h}+\varphi_{intz}T_{z} \end{array}$$ (10)

In Eqs (6)–(10), *T*_*int*_ is the time an individual spends on the internet; *φ*_*int*_ is the ratio of internet usage time to total time. *T*_*intw*_ is the time spent on the internet for work, and *φ*_*intw*_ is the proportion of work time dedicated to internet use. *T*_*inth*_ is the time using the internet in the health production process, *φ*_*inth*_ is the proportion of time within this process allocated to internet use. *T*_*intz*_ is other internet usage time, *φ*_*intz*_ is the proportion of other internet usage time dedicated to internet use. Becker postulates that the health level Hand other consumer goods *Z* follow a Leontief utility function, implying:

$$\begin{array}{c} H=\text{min}(\frac{M}{b_{m}},\frac{T_{h}}{t_{h}}) \end{array}$$ (11)

$$\begin{array}{c} Z=\text{min}(\frac{X}{b_{x}},\frac{T_{z}}{t_{z}}) \end{array}$$ (12)

In Eqs (11) and (12), *b*_*m*_ *b*_*x*_ *t*_*h*_ *t*_*z*_ represent the inputs per unit of production. Specifically, *b*_*m*_ is the amount of medical services required to produce one unit of health; *t*_*h*_ is the time taken to produce one unit of health; *b*_*x*_ is the quantity of X needed to produce one unit of *Z*; and *t*_*z*_ is the time consumed to produce one unit of Z. As the only market input for health production is medical services, *t*_*h*_ can be seen as the time individuals spend accessing these services, including travel, queuing for payments, waiting for appointments, etc. Under the constraint of minimizing production costs, the optimal input combination is:

$$\begin{array}{c} \frac{M}{b_{m}}=\frac{T_{h}}{t_{h}}=H \end{array}$$ (13)

$$\begin{array}{c} \frac{X}{b_{x}}=\frac{T_{z}}{T_{z}}=Z \end{array}$$ (14)

So:

$$\begin{array}{c} M=b_{m}H \end{array}$$ (15)

$$\begin{array}{c} T_{h}=t_{h}H \end{array}$$ (16)

$$\begin{array}{c} X=b_{x}Z \end{array}$$ (17)

$$\begin{array}{c} T_{z}=T_{z}Z \end{array}$$ (18)

Substituting Eqs (15)–(18) into (4) and (5) yields:

$$\begin{array}{c} (P_{m}b_{m}+Wt_{h})H+(P_{x}b_{x}+Wt_{z})Z=N+WT_{w} \end{array}$$ (19)

Under the income constraint of Eq (19), maximize the individual utility in Eq (1), the equilibrium condition is:

$$\begin{array}{c} \frac{U_{\hbar }}{U_{z}}=\frac{\pi_{h}}{\pi_{z}} \end{array}$$ (20)

In:

$$\begin{array}{c} \pi_{h}=P_{m}b_{m}+Wt_{h} \end{array}$$ (21)

$$\begin{array}{c} \pi_{z}=P_{x}b_{x}+Wt_{z} \end{array}$$ (22)

The economic meanings of Eq (20) is that the optimal quantity of health and other consumption for an individual occurs when the ratio of their marginal effects equals the ratio of their relative prices. The economic meanings of Eqs (21) and (22) is that the price of both not only encompasses the monetary value in the market but also includes the time cost associated with consuming the item. Thus, *π*_*h*_ can be viewed as the shadow price of health. Therefore, according to Eq (21), the cost incurred by an individual for each medical visit consists of two components: first, the monetary cost; second, the time cost. Based on Eqs (6) to (10), the Internet affects health costs through time cost *t*_*h*_, subsequently influencing health equilibrium. Combined with Eqs (10), (16) and (21), we have:

$$\begin{array}{c} t_{h}=\frac{T_{h}}{H}=\frac{T_{inth}H}{\varphi_{inth}} \end{array}$$ (23)

Let

$$\begin{array}{c} T_{inth}=\alpha T_{int} \end{array}$$ (24)

In Eq (24), *α* is the proportion of total internet time devoted to health production. Substituting into Eq (23), we have:

$$\begin{array}{c} t_{h}=\frac{T_{h}}{H}=\frac{\alpha T_{int}H}{\varphi_{inth}}=\frac{\alpha }{\varphi_{inth}}T_{int}H \end{array}$$ (25)

**Fig 1:**
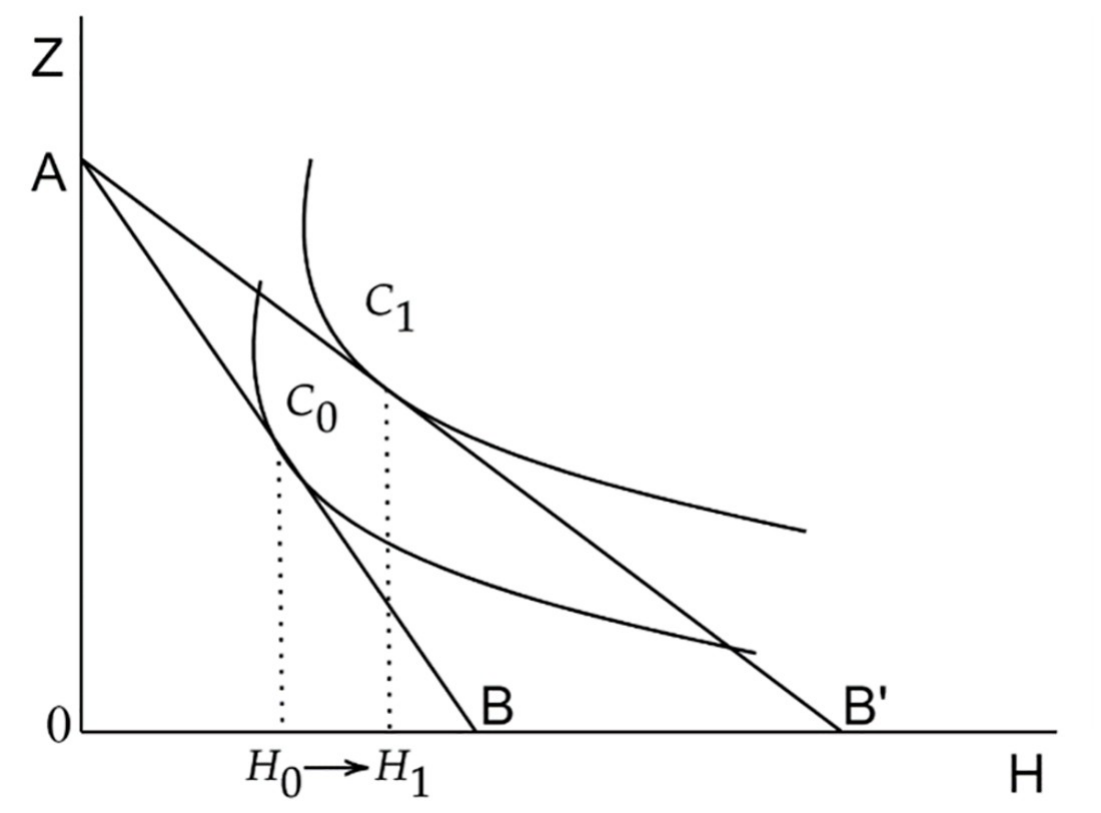
Changes in the individual’s budget line and indifference curve.

Analyzing Eq (25), where *T*_*i*_*nt* denotes individual internet usage time. Given the finite nature of an individual’s daily time, this can be considered a constant value. Based on Eqs (11) and (15), *H* can be viewed as an exogenous variable determined by *M* and *b*_*m*_. Hence, *t*_*h*_ is determined by $\frac{\alpha }{\phi_{\text{inth}\;}}$. Here, *α* is the fraction of total internet time dedicated to health production, and *φ*_*inth*_ is the rate of internet usage within the time allocated for health production. Thus, the meaning of $\frac{\alpha }{\phi_{\text{inth}\;}}$ can be interpreted as the reciprocal of the internet health production efficiency. If $\frac{\alpha }{\phi_{\text{inth}\;}}$ is smaller, the internet health production efficiency is higher. The time *t*_*h*_ needed to produce one unit of health is reduced, as depicted in Fig 1.

As shown in Fig 1, the use of the internet reduces the time cost of medical services, *t*_*h*_, leading to a decrease in the shadow price of health, *π*_*h*_. Consequently, the relative price of health to other consumer goods also declines, causing the budget line in Fig 1 to pivot outward from AB to AB’. This manifests in two ways: First, there’s a substitution effect. As the relative price of health drops, individuals will increase their demand for health. Second, there’s an income effect. Since health is not considered an inferior good, the income effect is positive. With the effective increase in income, the demand for health naturally rises. From the analysis above, a reduction in *t*_*h*_ leads to an increase in an individual’s health demand, influenced by both the substitution and income effects. As depicted in the figure, this shifts from *H*_0_ to *H*_1_.

By incorporating the internet into Becker’s health demand model, we observe that it primarily affects people’s health through internet health productivity. Internet health productivity refers to the amount of internet time required to produce one unit of health time, which is a theoretical concept. In real life, other pathways of influence may exist. The next section will analyze the potential mechanisms of influence that may exist in daily life.

### Mechanism analysis

First, Using the internet has mitigated information asymmetry concerning health. Firstly, it has narrowed the informational divide between doctors and patients. This gap is pronounced due to the healthcare industry’s monopolistic hold on health information, with top-tier hospitals having a distinct advantage in both information and resources. people often place their trust in well-known hospitals from other regions, displaying skepticism towards local ones. However, with internet access, diversified information channels significantly influence residents’ medical decisions (Liu Chen and Zhou Xianghong, 2017) [26]. They can collect essential details ahead of their consultations, allowing them to gauge their medical conditions and choose appropriate hospitals for treatment (Mcmullan, 2006) [10], thus reducing the doctor-patient information gap. Secondly, between people, information asymmetry is evident as health information access varies based on educational backgrounds, ages, or living conditions. After engaging with the internet, people benefit from a transparent and equitable information source. It serves as a platform for communication, alleviating feelings of loneliness and depression among people (Lagoe and Atkin, 2015) [4], and doubles as a tool for information search, helping people find health-promoting details (Dutta-Bergman, 2004) [6].

Second, Using the internet reduced the costs associated with accessing health services, encompassing both time and monetary expenses. The reduction in time cost stems from the departure from traditional offline consultations. Internet-enabled features such as online appointment booking, e-consultations, and mobile payment systems have greatly enhanced the efficiency with which people access healthcare. This has reduced the number of visits to hospitals, consequently lowering the time cost of healthcare. As for the monetary aspect, cost savings arise from paperless test results and other reduced medical service expenses, as well as the convenience it provides for insurance claims. For instance, data from Ningbo, China, shows that after the adoption of digital imaging, costs dropped from 22 yuan for traditional films to 8 yuan for digital reports. This single change saved patients in the city over 100 million yuan in medical expenses in 2018. Furthermore, the digitization of health insurance, along with the introduction of services like cross-regional medical consultations and swift insurance claim processing, has bridged the gap between medical institutions and insurance companies. This has increased the success rate and efficiency of insurance claims for patients. Hence, the use of the internet by people has reduced both the time and monetary costs of healthcare, making medical services more accessible and contributing to improved public health.

Third, Using the internet the internet has enhanced their exposure to healthier environments, both in the workplace and in daily life. On one hand, the internet has driven the transformation of traditional industries. The labor market is gradually shifting from traditional roles to those requiring newer technological skills. Tasks that previously exposed workers to harsh conditions are now being replaced by human-computer interactions or artificial intelligence, allowing people to work in more comfortable settings and thereby increasing their exposure to healthier work environments. On the other hand, the proliferation of internet-connected home devices and digital tools has amplified exposure to healthier living environments. Wearable tech can continuously monitor vital health metrics, while smart car air quality systems can measure and filter out outdoor pollutants in real-time. Internet-enabled household devices, such as robotic vacuums and dishwashers, can assume a significant portion of domestic chores, creating a more favorable living environment. Therefore, the integration of the internet into daily life has expanded residents’ exposure to healthier environments, contributing to improved public health.

Fourthly, Using the internet the internet should be approached with moderation. Prolonged immersion in virtual environments can impact psychological health, and discerning accurate information amid the deluge can be challenging. Numerous scholars have found that excessive or inappropriate internet usage can lead to psychological issues such as anxiety and feelings of emptiness (Brenner, 1997; Allcott et al., 2020) [27, 28]. In regions like China and other parts of Asia, there’s a heightened susceptibility to internet addiction (Dong et al., 2012) [29]. While the internet indeed offers a vast information repository, the ease of content dissemination has resulted in platforms being inundated with information of varying quality. If people struggle to differentiate valuable information from misinformation, they risk making decisions detrimental to their health.

Based on the above theoretical analysis, the internet promotes residents’ health levels in three main ways:1, Reducing information asymmetry related to health;2, Lowering the costs associated with acquiring health services; 3, Enhancing exposure to healthier environments. However, there are conditions for moderation when using the internet: Engaging with the internet at an appropriate intensity; and differentiating accurate information from the myriad available online. Only under these conditions can the use of the internet optimally promote health levels. We hypothesize that internet use affects residents’ health levels. However, the impact varies across different health aspects and among different individuals.

## Data and empirical strategy

In this section, we present the setup of the difference-in-differences model, detail the data sources and variable selections, and provide descriptive statistics for the data.

### Model

To investigate the impact of the internet on residents’ health, based on the mechanism analysis discussed, we established a multi-time point Difference-in-Differences (DID) model with both time and regional fixed effects. This is a commonly used econometric method for evaluating the effects of policies or interventions. In this study, we consider internet use as an intervention. The treatment group consists of individuals who use the internet, while the control group consists of those who do not.

Step 1: Propensity Score Matching (PSM) involves creating a propensity score model to match individuals in the treatment and control groups with similar characteristics on observed variables. This approach helps eliminate the influence of other factors, isolating the effect of the intervention.

Step 2: Difference in Differences (DID) is used after verifying the parallel trends assumption. This assumption states that, in the absence of the intervention, the trends for the treatment and control groups should be similar. By comparing the differences between the two groups before and after the intervention, we can isolate the effect of the intervention from time-related or other variable influences.

The advantage of using the PSM-DID method in our study is that internet use is not a standard natural experiment and may involve various interfering factors. The traditional DID method might suffer from self-selection bias. However, using PSM allows us to match each treated sample with a specific control sample, making the quasi-experiment approximately random. By examining the health level changes of people before and after their household’s broadband internet adoption, we aim to identify the impact of the internet on residents’ health conditions. The model is as follows:

$$\begin{array}{c} {\text{PhyHealth}}_{it}=\alpha +\beta \;{\text{treat}}_{i}\times {\text{int}}_{it}+\sum \gamma X_{i,t}+\delta_{it}+\mu_{it}+\varepsilon_{it}\;\left(\text{modle}\;1\right) \end{array}$$

$$\begin{array}{c} {\text{PsyHealth}}_{it}=\alpha +\beta \;{\text{treat}}_{i}\times {\text{int}}_{it}+\sum \gamma X_{i,t}+\delta_{\text{it}}+\mu_{\text{it}}+\varepsilon_{\text{it}}\;\left(\text{modle}\;2\right) \end{array}$$

In Model1, PhyHealth_it_ is the physical health status of resident *i* in year *t*. In Model2 PsyHealth_it_ is the psychological health status of resident *i* in year *t*. treat_it_ is a dummy indicating whether resident *i* belongs to the treatment group. If the resident engaged in digital activities during the experimental period, ‘treat’ equals 1; otherwise, it’s 0. int_it_ is a dummy variable indicating whether household of resident i uses the internet in year t, with a value of 1 if they did, and 0 otherwise. *β* is the treatment effect, signifying the health change after people engage in digital activities. *X*_*i*,*t*_ is a set of control variables, encompassing individual, household, and regional-level controls. *δ*_*it*_ and *μ*_*it*_ respectively signify time and regional fixed effects, and *ε*_*it*_ denotes the random error term.

### Data

Our study sample comes from the China Health and Retirement Longitudinal Study (CHARLS). This survey is a large-scale interdisciplinary project hosted by the National School of Development at Peking University and executed by the China Social Science Survey Center and the Communist Youth League of Peking University. The aim is to collect high-quality microdata representing individuals and households aged 45 and above in China to analyze the issues related to population aging, promote interdisciplinary research, and provide a scientific basis for formulating and improving related policies in China.

The CHARLS questionnaire design draws on international experience, including the Health and Retirement Study (HRS) in the United States, the English Longitudinal Study of Ageing (ELSA), and the Survey of Health, Ageing and Retirement in Europe (SHARE). The project employs a multi-stage sampling method, with PPS sampling at the county/district and village/community stages. CHARLS pioneered the use of electronic mapping software (CHARLS-GIS) to create village-level sampling frames using mapping methods.

The CHARLS questionnaire includes sections on personal basic information, family structure and economic support, health status, physical measurements, healthcare utilization and insurance, employment, retirement and pensions, income, consumption, assets, and basic community information. The survey began in 2011, covering 150 county-level units and 450 village-level units, with about 17,000 individuals from 10,000 households tracked every two to three years. This database covers most regions nationwide and includes comprehensive information on residents’ physical health, lifestyle, and medical treatment, providing ample samples and suitable indicators for our quasi-natural experiment design.

We utilize data from CHARLS 2013, CHARLS 2015, and CHARLS 2018, spanning three survey years. The empirical approach employed is a two-way fixed-effects difference-in-differences estimation over multiple time points. To ensure continuity in observation targets, the sample scope has been limited to those who responded to the digital activity-related questions across all three survey years. After excluding missing values for the main variables, the final sample comprises data from 151 regions, 7,408 households, yielding a total of 32,157 valid samples. During the empirical analysis, individual and household data are sourced from the CHARLS survey database, while regional data are derived from the “China City Statistical Yearbook”, the “China Regional Statistical Yearbook”, and the EPS data platform.

### Variable

#### Explained variable

Drawing upon the research by Wang Yuzhe et al. (2020) [30] and Wang Yuzhe and Luo Nengsheng (2020) [31], residents’ health is measured in terms of physical health and psychological health. Based on McClellan (1998) [32], physical health is gauged through two sub-indicators: acute and chronic diseases. These indicators are derived from the HEALTH STATUS section of the CHARLS survey data. psychological health, referencing the study by Wen Xingxiang et al. (2017) [33], is evaluated through three sub-indicators: episodic memory, cognitive function, and self-assessed depression. These are sourced from the COGNITION & DEPRESSION section of the CHARLS survey data. The entropy method is employed to determine the weights of each sub-indicator, providing an objective representation of the distinctions and significance among them. This results in scores for both physical and psychological health. Detailed metric specifications and weights are presented in Table 1.

**Table 1:** Construction of resident health indicator system.

Primary Indicator	Secondary Indicator	Indicator Content	Attribute	Weight
Physical	Acute Disease	Respondents reported having conditions such as heart disease, stroke, or cancer. Each condition is scored one point. Score range: [0, 3].	Negative	0.666
	Chronic Disease	Respondents indicated the presence of hypertension, diabetes or high blood sugar, chronic lung disease, arthritis or rheumatism, abnormal blood lipids, liver disease, kidney disease, stomach or other digestive disorders, asthma, etc. Each condition is given one point. Score range: [0, 9].	Negative	0.334
Psychological	Situational Memory	The number of times respondents can immediately or later recall the correct phrases. Score range: [0, 20].	Positive	0.293
	Cognitive Awareness	Instances where respondents can correctly calculate, recall dates, duplicate graphics, etc. Score range: [0, 9].	Positive	0.339
	Depression Self-assessment	Respondents self-assess their depression level, including 10 related questions. The sum of the points from the selected options is the depression self-assessment score. Score range: [0, 30].	Negative	0.368

For example, in the acute diseases section of physical health, respondents report the presence of heart disease, stroke, cancer, etc. Each disease is scored as one point, with a score range of [0, 3]. Specific questions from the CHARLS questionnaire include:

DA007_7: “Have you been diagnosed with Heart attack, coronary heart disease, angina, congestive heart failure, or other heart problems by a doctor?” 1. Yes; 2. No.

DA007_8: “Have you been diagnosed with Stroke by a doctor?” 1. Yes; 2. No.

DA007_4: “Have you been diagnosed with Cancer or malignant tumor (excluding minor skin cancers) by a doctor?” 1. Yes; 2. No.

#### Explanatory variable

Core explanatory variable: The interaction term treat × Digital. This reflects the difference in health changes before and after participating in digital life between people in the experimental group and those in the control group. The data for Digital is sourced from the HOUSING CHARACTERISTICS section of the CHARLS survey, specifically question I025, “Does your household have broadband internet access?” If respondents answer “YES”, it is coded as 1, indicating participation in digital life. If the answer is “NO”, it’s coded as 0, denoting non-participation in digital life.

Control variables: Based on Grossman’s health demand theory (Grossman, 1972) [34], factors affecting residents’ health mainly come from three aspects: individual, family, and region. Building on previous studies and considering data availability and model integrity, this paper has selected the following control variables. The definitions and descriptive statistics for each variable are presented in Tables 2 and 3.

**Table 2:** Meaning of main variables.

Type	Name	Symbol	Definition and Assignment
Individual	Physical Health	PhyHealth	Based on the entropy weight method, a higher score indicates better physical health.
	Psychological Health	PsyHealth	Based on the entropy weight method, a higher score indicates better psychological health.
	Self-assessed Health	SelfHealth	From the CHARLS survey, a higher score suggests the respondent perceives themselves as healthier, with a score range of [0, 5].
	Age	Age	The age of the respondent for that year.
	Gender	Sex	The gender of the respondent: Male = 1, Female = 0.
	Marital Status	marriage	Marital status of the respondent: Cohabiting or married = 1; Single, separated, divorced, or widowed = 0.
	Urban/Rural Residency	residence	Urban residence = 1; Rural residence = 0.
	Sleep Quality	Sleep	Frequency with which the respondent felt restless in their sleep over the past week; a higher score indicates more frequent restlessness.
	Health Insurance Coverage	Insure	Having health insurance = 1; No health insurance = 0.
Family	Number of Toilets	lavatory	Number of toilets in the respondent’s household.
	Access to Running Water	Water	Access to running water = 1; No access to running water = 0.
	Internet	Int	Broadband installed = 1; No broadband installed = 0.
Regional	Economic Status	Gdp	The logarithm of the local average GDP.

**Table 3:** Descriptive statistics.

Name	Number	Standard	Deviation	Min	Max
PhyHealth	32157	−0.115	0.135	−0.866	0.001
PsyHealth	32157	0.239	0.179	−0.368	0.632
SelfHealth	32157	3.035	0.972	1	5
Int	32157	0.289	0.453	0	1
Age	32157	61.2	9.373	15	108
Sex	32157	0.478	0.5	0	1
education	32157	3.418	1.899	1	9
marriage	32157	0.877	0.328	0	1
residence	32157	0.658	0.474	0	1
Sleep	32157	2.076	1.214	1	4
Insure	32157	0.947	0.224	0	1
lavatory	32157	1.023	0.836	0	9
Water	32157	0.751	0.432	0	1
Gdp	32157	16.754	0.86	14.568	19.54
People	32157	5.846	0.956	2.351	7.739
Medical	32157	9.189	0.694	6.795	11.455
sewage	32157	0.863	0.133	0.235	1
rubbish	32157	0.913	0.15	0.192	1

## Empirical results

In this section, we conduct a parallel trends test preceding the DID model, illustrate the PSM procedure, run the basic regression for the model, discuss potential endogeneity concerns, and perform robustness checks on the results.

### Parallel trend test

The difference-in-differences (DID) method can address endogeneity issues caused by sample selection. For empirical analysis, it requires the parallel trends assumption to hold; namely, in the absence of experimental shocks, both the treatment and control groups would follow the same time trends. Additionally, changes in different control variables should be almost consistent, except for the experimental variable.

**Fig 2:**
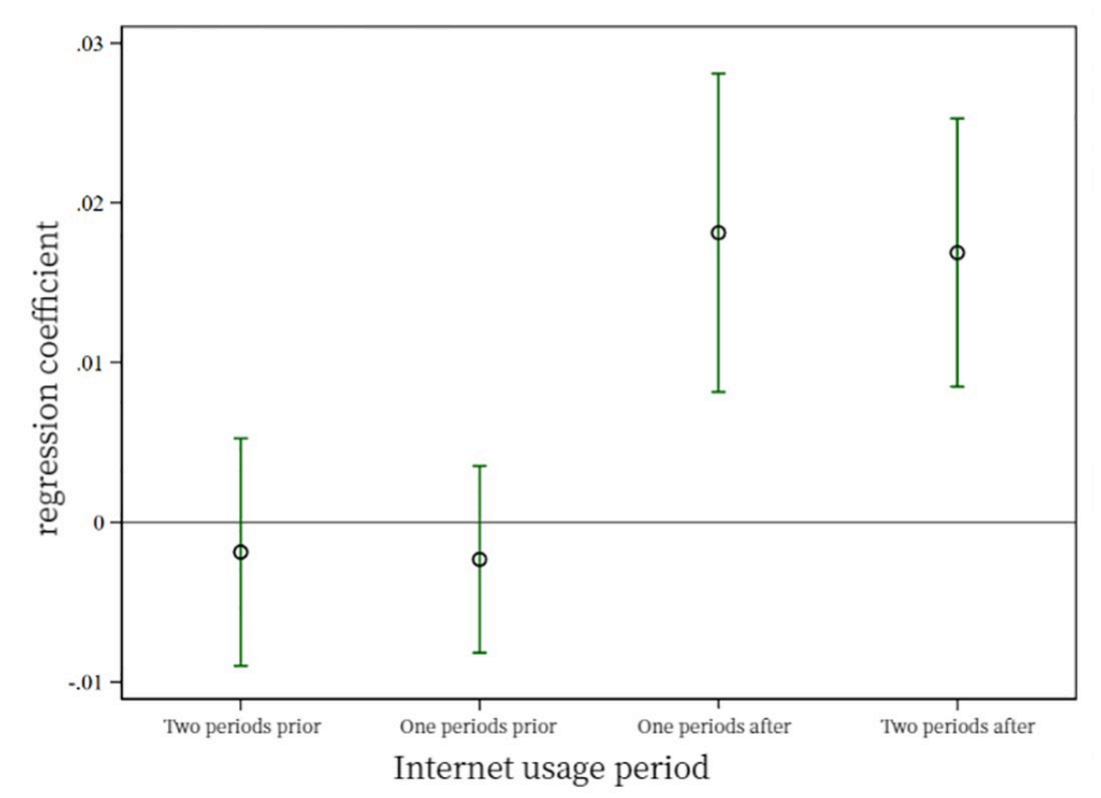
Physical health parallel trend test.

Fig 2 tests the parallel trends for physical health. The regression coefficients intersect with the horizontal axis for the two periods before people engaged in digital life, indicating insignificance. However, for the period after engagement, the coefficients cross the axis and become significant, suggesting that physical health satisfies the parallel trend assumption.

**Fig 3:**
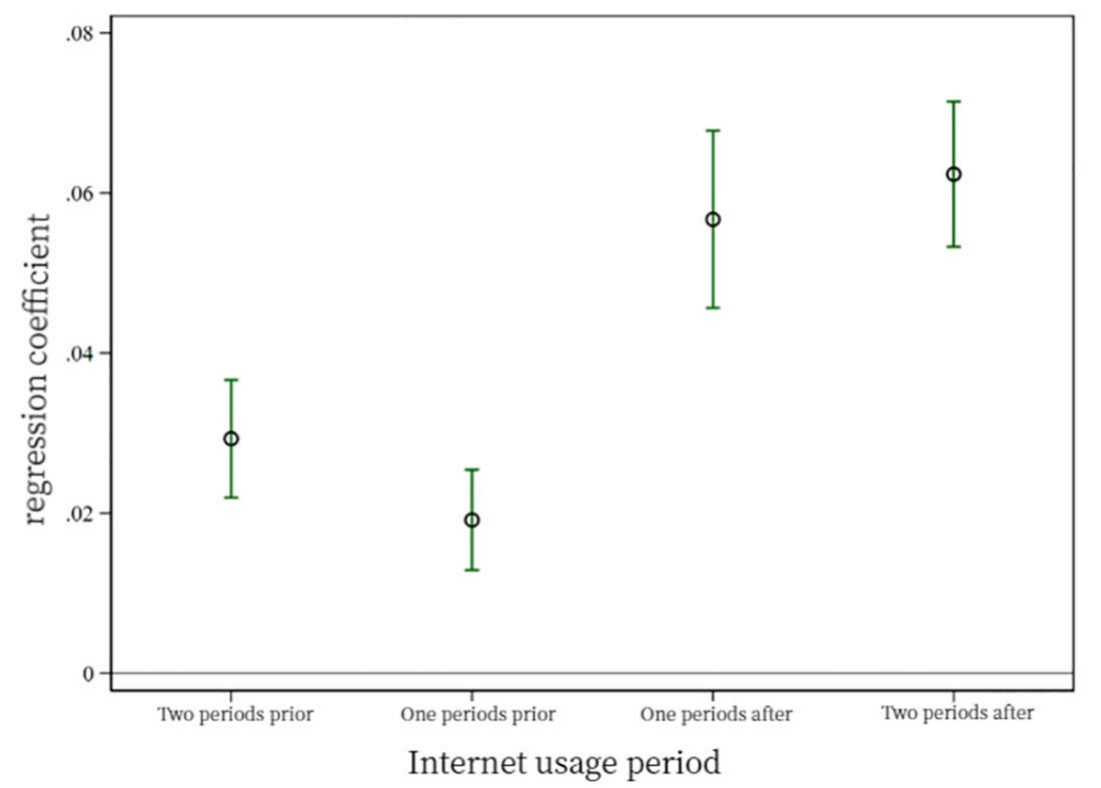
Psychological health parallel trend test.

Fig 3 tests the parallel trends for Psychological Health. Although the regression coefficients do not intersect with the horizontal axis for the two periods both before and after the engagement in digital life, indicating that, statistically, psychological health doesn’t satisfy the parallel trends test. However, observing the graph shows significant trend changes in the coefficients for the two periods after engagement. These changes are relatively consistent, leading this study to believe that there’s still some level of parallel trend regarding the impact of digital life on psychological health.

### Propensity score matching

**Fig 4:**
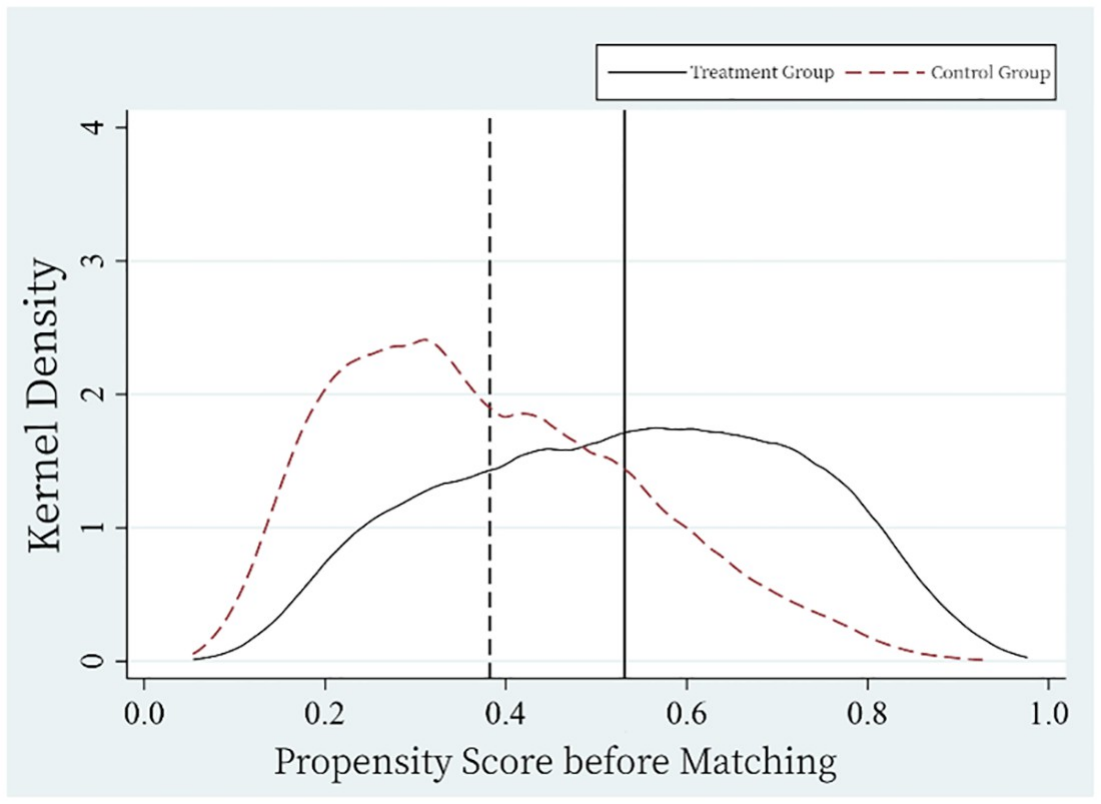
Propensity score before matching.

**Fig 5:**
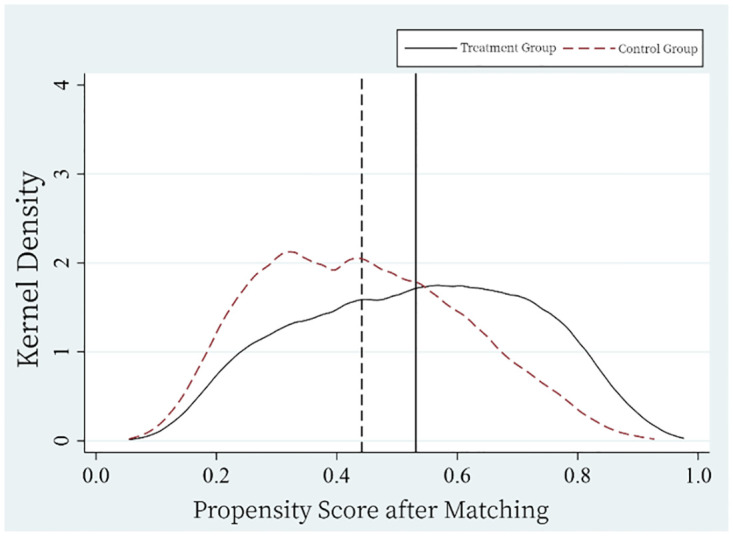
Propensity score after matching.

To further address observable and unobservable differences between the treatment and control groups, mitigate potential biases from non-parallel trends and selection, this study employed Propensity Score Matching (PSM). The nearest neighbor matching was adopted using a one-to-one matching protocol, where each treated sample was matched to at most one control sample. The caliper was set at 0.05. Fig 4 presents the propensity scores before matching, while Fig 5 shows them after matching. Notably, there was a significant discrepancy in kernel density curves between the two groups before matching, which was considerably reduced after matching, bringing the mean propensity scores closer together. Thus, through PSM, the gap between the treatment and control groups was reduced, enhancing the robustness of the difference-in-differences estimates.

### Baseline regression

Tables 4 and 5 present the stepwise regression results evaluating the impact of internet use on physical and psychological health, respectively. After propensity score matching, to prevent large disparities in the weight of control group samples, we replicated matched samples in the control group using frequency-weighted regression, increasing the sample size to 37,372. Column (1) includes only the “Internet” variable, controlling for time and region. Column (2) displays results with individual controls, column (3) with individual and family controls, and column (4) with individual, family, and regional controls. In both Tables 4 and 5, the regression coefficients for “Internet” are consistently positive, suggesting significant health improvements for people who started using the internet. In column (4), the estimated coefficient for the internet’s effect on physical health is 0.0111, while it’s 0.0266 for psychological health, indicating that internet use has a greater positive impact on psychological health compared to physical health.

**Table 4:** Physical health baseline regression results.

	Physical Health			
	(1)	(2)	(3)	(4)
Internet	0.0276***	0.0117***	0.0112***	0.0111***
	(7.7281)	(3.3165)	(3.1967)	(3.1643)
Individual Controls	NO	YES	YES	YES
Family Controls	NO	NO	YES	YES
Regional Controls	NO	NO	NO	YES
Time Fixed Effects	YES	YES	YES	YES
Region Fixed Effects	YES	YES	YES	YES
N	37372	37372	37372	37372
R^2^	0.1511	0.1984	0.1989	0.1991

The value in parentheses is the T-value;

* p<0.10,

** p<0.05,

*** p<0.01.

the same applies below.

**Table 5:** Psychological health baseline regression results.

	Physical Health			
	(1)	(2)	(3)	(4)
Internet	0.0256***	0.0262***	0.0266***	0.0266***
	(6.0949)	(6.7420)	(6.8528)	(6.8533)
Individual Controls	NO	YES	YES	YES
Family Controls	NO	NO	YES	YES
Regional Controls	NO	NO	NO	YES
Time Fixed Effects	YES	YES	YES	YES
Region Fixed Effects	YES	YES	YES	YES
N	37372	37372	37372	37372
R^2^	0.1647	0.3664	0.3666	0.3668

### Endogeneity

We recognize potential endogeneity issues. First, there’s possible reverse causality: the internet’s health benefits might be driven by healthier individuals more likely to use it, while those in poor health might not. Yet, internet usage is gauged at the household level, where individual health has limited influence, but it’s crucial not to overlook. Second, while most control variables are derived from the CHARLS questionnaire, some micro or macro factors might be missing due to data limitations. Third, measurement errors arise in CHARLS as responses are subjective, and vast data could lead to oversights. To address endogeneity, the study adopts an instrumental variable approach, using China’s 2015 “Internet+” directive. This policy, promoting internet integration with traditional industries, aligns with residents’ internet use (satisfying relevance) and doesn’t link to health otherwise (ensuring exogeneity). Therefore, a two-stage least squares method is used with values assigned based on policy timelines. Due to multicollinearity from policy dummies, only regional effects are fixed. As Table 6 shows, both physical and psychological health coefficients remain significantly positive, confirming the robust influence of the internet on residents’ health after considering endogeneity.

**Table 6:** Instrumental variable regression results.

	Physical Health	Psychological Health
Internet	0.0418***	0.0357***
	(4.299)	(3.37)
Individual Controls	YES	YES
Family Controls	YES	YES
Regional Controls	YES	YES
Time Fixed Effects	NO	NO
Region Fixed Effects	YES	YES
N	37372	37372
R^2^	0.122	0.385

### Robustness test

To enhance the rigor of our conclusions, we conducted robustness tests in three dimensions: 1) In the 1 column, we adjusted the dependent variable, initially computed using the entropy method for physiological and psychological health indices, to respondents’ self-rated health scores to address potential biases from measurement indicators; 2) In the 2 and 4 column, we incorporated control variables, specifically an urban digital economy metric from Zhao Tao et al. (2020) [35], given the potential health impact of local digital economic progression; 3) In the 3 and 5 column, we modified model estimation conditions, shifting from one-to-one to one-to-two nearest neighbor matching in propensity score matching, thereby increasing the sample size for regression. The consistent and significant results from Table 7 underscore the robustness of our conclusions.

**Table 7:** Robustness test results.

	Self-rated Health	Physiological Health		Psychological Health	
	(1)	(2)	(3)	(4)	(5)
Internet	0.1089***	0.0059*	0.0089***	0.0220***	0.0252***
	(4.7351)	(1.7538)	(2.8745)	(5.6664)	(7.1084)
Ind Controls	YES	YES	YES	YES	YES
Fam Controls	YES	YES	YES	YES	YES
Reg Controls	YES	YES	YES	YES	YES
Time Fixed	YES	YES	YES	YES	YES
Region Fixed	YES	YES	YES	YES	YES
N	37372	37196	39397	37196	39397
R^2^	0.1603	0.1902	0.1818	0.3637	0.3486

## Further analysis

In this section, we first conduct empirical tests on the pathway analyses presented, including information asymmetry, medical costs, and exposure to health environments. Subsequently, we delve into heterogeneity analyses, encompassing varying internet usage content, intensity of internet use, and factors like age, medical insurance, and local digital economic levels.

### Mechanism verification

The section’s theoretical analysis indicates that the internet’s impact on health is primarily realized through the reduction of information asymmetry, the decrease in health acquisition costs, and the enhancement of exposure to healthful environments. Table 8 employs grouped regression based on individuals’ decisions to seek medical treatment locally or elsewhere, aiming to examine the internet’s role in reducing information asymmetry. With the progression of medical technology, local healthcare can cater to most residents’ needs, barring complex cases. In China, local medical services come with added benefits such as higher insurance reimbursement and commute convenience. However, individuals, lacking comprehensive treatment information, often favor superior out-of-town hospitals, sidelining local institutions. The internet provides a platform for individuals to compare hospitals across regions, facilitating informed decisions tailored to their conditions. Results from Table 8 suggest that for those opting for out-of-town medical services, the internet doesn’t affect their health. Conversely, for local care seekers, the internet has a positive bearing on both physical and psychological health. This underscores that with the internet, individuals can make discerning choices, curbing the penchant for out-of-town treatments, thereby addressing information asymmetry, optimizing medical resource allocation, and consequently enhancing overall health.

**Table 8:** Verification of information asymmetry mechanism.

	Physical Health		Psychological health	
	Out-of-Town Medical Treatment	Local Medical Treatment	Out-of-Town Medical Treatment	Local Medical Treatment
Internet	0.0128	0.0156*	0.0281	0.0250***
	(0.7792)	(1.9358)	(1.4676)	(2.9871)
Control Variables	YES	YES	YES	YES
Fixed Effects	YES	YES	YES	YES
N	829	2955	829	2955
R^2^	0.1175	0.1028	0.3485	0.4035

Table 9 examines the mechanisms of health cost and environmental exposure to health. Column (1) focuses on the mechanism of health costs, operationalized as the time cost associated with healthcare. This is derived from the CHARLS survey, which tracks the frequency of visits to clinics or hospitals. A higher frequency implies elevated time costs. The results show a significant negative relationship between internet use and health time costs, indicating the internet reduces these costs. As per Becker’s health demand model discussed earlier, a decrease in health time costs results in a reduction in the relative price of health, leading to higher health consumption and improved health outcomes. Column (2) tests the mechanism of health environment exposure. The exposure metric, sourced from CHARLS, is based on interviewer ratings of respondents’ living conditions. A higher score denotes a cleaner, more hygienic environment. The findings reveal a significant positive correlation between internet usage and living environment quality. Greater exposure to healthier environments reduces contact with harmful substances and pollutants, while also promoting psychological health. Thus, internet usage enhances exposure to healthier environments, subsequently improving overall health.

**Table 9:** Verification of health cost and health environment exposure mechanism.

	Health Cost	Health Environment Exposure
	(1)	(2)
Internet	−0.1476*	0.3082***
	(−1.6709)	(14.5925)
Control Variables	YES	YES
Fixed Effects	YES	YES
N	5978	31395
R^2^	0.023	0.1558

### Internet content heterogeneity

Studies have indicated that different online behaviors yield varied impacts on individuals. For instance, utilizing digital currencies can enhance daily efficiency, while activities like online chatting and watching news videos can alleviate mood. Using data from the CHARLS survey, this study categorizes respondents’ daily internet engagement into three types:1. Economic internet activities include responses related to financial management, stock trading, and digital currency payments. 2. Social internet activities encompass responses about online chatting and making friends. 3. Entertainment internet activities comprise responses about browsing news, watching videos, and other entertainment-related behaviors.

Table 10 presents the empirical results of the health impact of these three internet activity categories. Only economic internet activities showed a significant positive impact on health, suggesting that online economic behaviors can enhance people’s health levels. As discussed earlier, the convenience brought about by online transactions significantly boosts efficiency in daily work, lifestyle, and medical processes. Hence, in the heterogeneity analysis of internet engagement methods, only economic online activities yield beneficial effects on people’s health.

**Table 10:** Internet content heterogeneity.

	Physical Health			Psychological health		
	(1)	(2)	(3)	(4)	(5)	(6)
Economic	0.0400***			0.0333***		
	(5.5074)			(5.8814)		
Social		−0.0039			0.0026	
		(−0.5213)			(0.4509)	
Entertainment			−0.0179			0.0047
			(−1.5398)			(0.4692)
Control Variables	YES	YES	YES	YES	YES	YES
Fixed Effects	YES	YES	YES	YES	YES	YES
N	2335	2335	2335	2335	2335	2335
R^2^	0.0591	0.0431	0.0441	0.2665	0.2527	0.2527

In the preceding theoretical analysis, we posited that internet usage should be maintained at a moderate intensity. In this section, we investigate whether different intensities of internet usage lead to diverse health outcomes. Drawing from the CHARLS survey, we categorize responses based on the frequency of internet use. We distinguish internet usage into two intensities: high and low, with high intensity coded as 1 and low intensity as 0. Table 11 presents the regression results for heterogeneity in internet usage intensity. While internet intensity does not exhibit a significant impact on physical health, it shows a pronounced negative effect on psychological health. Relative to low intensity, high-intensity internet usage deteriorates individuals’ psychological health. Although the internet can offer relaxation and pleasure through entertainment and socializing, excessive or even addictive usage may exacerbate feelings of emptiness and loneliness, potentially leading to addiction or detachment from reality. Thus, high-intensity internet usage is not conducive to individuals’ psychological health.

**Table 11:** Heterogeneity in internet usage intensity.

	Physical Health	Psychological health
Internet	0.0256	-0.0259*
	(0.2576)	(-1.6804)
Control Variables	YES	YES
Fixed Effects	YES	YES
N	2125	2125
R^2^	0.0958	0.1021

Tables 12 and 13 presents heterogeneity tests for age, gender, economic status, health insurance status, and regional digital economy development. For age heterogeneity, results indicate that individuals aged 60 and above experience significant positive effects on both physical and psychological health from internet usage. Conversely, those under 60 only benefit mentally. This heterogeneity can be attributed to the generally stable and high physical health levels of those under 60, rendering minimal impact from internet usage. As individuals age, however, physical health challenges emerge. For those aged 60 and over, the internet effectively alleviates these challenges, enhancing their health.

**Table 12:** Additional heterogeneity tests.

Groups		Physical Health	Psychological health
Age 60 and above	Internet	0.0135***	0.0225***
		(3.9759)	(5.8912)
	Control Variables Fixed Effects	YES	YES
	N	17718	17718
Age below 60	Internet	0.0046	0.0230***
		(1.4986)	(6.2280)
	Control Variables Fixed Effects	YES	YES
	N	14439	14439

**Table 13:** Additional heterogeneity tests.

Groups		Physical Health	Psychological health
Male	Internet	0.0115***	0.0099*
		(3.2987)	(1.7026)
	Control Variables Fixed Effects	YES	YES
	N	18564	18564
Female	Internet	0.0094***	0.0209***
		(2.8741)	(5.3592)
	Control Variables Fixed Effects	YES	YES
	N	13593	13593
Good Economic status	Internet	0.0249***	0.0198***
		(3.5543)	(6.9014)
	Control Variables Fixed Effects	YES	YES
	N	8457	8457
Poor Economic status	Internet	0.0236	0.0113*
		(1.1187)	(1.7744)
	Control Variables Fixed Effects	YES	YES
	N	3722	3722
With Health Insurance	Internet	0.0097***	0.0216***
		(4.0201)	(7.7437)
	Control Variables Fixed Effects	YES	YES
	N	30448	30448
Without Health Insurance	Internet	0.0157	0.0303**
		(1.6372)	(2.2418)
	Control Variables Fixed Effects	YES	YES
	N	1709	1709
High Digital Economy	Internet	0.0115***	0.0216***
		(3.8248)	(6.2296)
	Control Variables Fixed Effects	YES	YES
	N	16890	16890
Low Digital Economy	Internet	0.0062*	0.0255***
		(1.7244)	(6.2296)
	Control Variables Fixed Effects	YES	YES
	N	15267	15267

The gender heterogeneity analysis shows that internet use significantly improves both physical and psychological health for men, although the improvement in psychological health is less significant. For women, internet use significantly enhances both physical and psychological health. Comparing the gender differences, the beneficial effect of internet use on psychological health is approximately twice as high for women as for men. This may be because women are more emotional and enjoy entertainment and emotional content on the internet, which significantly impacts their psychological well-being.

The economic status heterogeneity analysis reveals that residents with better economic status experience significant improvements in both physical and psychological health after using the internet. In contrast, residents with poorer economic status only see improvements in psychological health. This may be because residents with better economic status have a higher efficiency in converting internet benefits into health improvements.

Regarding health insurance, individuals with insurance exhibit significant health improvements when using the internet, whereas those without insurance do not see enhanced physical health. With insurance, lower medical service costs ensure better health outcomes. Without insurance, despite some cost reductions from internet utilization, the disparities compared to insured rates are substantial, preventing the internet from significantly boosting physical health.

For digital economy development heterogeneity, we employ city-level metrics from Zhao Tao et al. (2020) [35], classifying regions into higher or lower digital economy development based on medians. Findings suggest that regions with a more advanced digital economy enhance residents’ physical health more effectively. A higher digital economy indicates superior digital infrastructure, better internet usage quality, and access to premium digital services, maximizing the internet’s benefits. Consequently, internet usage in digitally advanced areas fosters greater physical health improvements.

## Discussion

In this study, we explore the impact of internet access on physical and psychological health, identifying the pathways and heterogeneous effects involved. Our baseline analysis indicates that internet access benefits both physical and psychological health, aligning with the findings of scholars like Karyotaki et al. (2021) [36]. We propose three mechanisms—information asymmetry, healthcare costs, and exposure to health environments—all empirically validated, echoing the conclusions of Han et al. (2020) [37], Dimitrov (2016) [38], and Li X et al. (2017) [39].

Heterogeneity analysis, particularly regarding age, presents findings divergent from some studies. We observe that individuals over 60 experience positive health impacts from internet access, contrary to Xie et al. (2021) [16], who suggest internet use may exacerbate depression symptoms among the elderly. In the gender heterogeneity analysis, we observed that the impact of internet use on health varies by gender. This aligns with Bao and Huang’s studies (2020, 2021, 2022a, 2022b, 2023) on the effects of technology on gender differences and equality. [40–44] Moreover, Zhao et al. (2022) [45] indicate that the elderly face barriers in internet usage. These discrepancies may stem from our focus on household internet access as the explanatory variable, rather than individual usage. Despite obstacles faced by the elderly in using the internet, household access enables family members to assist them in seeking health information and medical aid, thereby improving their health outcomes.

Our research stands out in several ways. We utilize the CHARLS survey to objectively score physical health. Additionally, we validate multiple pathways between the internet and health, introducing the concept of increased exposure to health environments via the internet. In our heterogeneity analysis, we consider the level of digital economy development, revealing that the impact of the internet on health is significantly influenced by digital infrastructure, suggesting new directions for future health improvements.

However, limitations exist due to data constraints, leading to a somewhat narrow focus in parts of our study. For instance, in examining healthcare costs, we only consider time costs, omitting monetary costs. Our exploration of health environment exposure is limited to the home setting, excluding work (Li X et al., 2017) [39] and medical environments (Newman et al., 2019) [46]. Furthermore, our heterogeneity analysis lacks consideration of factors like gender (Nam and Han, 2019) [47], education (Bol et al., 2018) [48], and healthcare market conditions (Li et al., 2016) [49]. Future research should aim to address these gaps. Additionally, we identify several future research directions. These include exploring the long-term effects of internet use on health, examining the role of digital literacy in moderating health outcomes, and conducting similar studies in different cultural contexts for comparative analysis. These additions aim to provide clear directions for future research based on our findings.

## Conclusions and recommendations

Based on the Becker health demand model, we analyzes how internet use impacts residents’ health by reducing information asymmetry, lowering healthcare costs, and increasing exposure to health environments. Through this model, we explore the specific mechanisms by which the internet enhances health levels. We believe this theoretical framework provides new perspectives and significant theoretical contributions to understanding the impact of internet use on health. Empirically, using data from the CHARLS survey, we employ a propensity score matching difference-in-differences approach to examine the internet’s impact on health. The findings indicate favorable effects of internet usage on both physical and psychological health. The validation of mechanisms confirms the existence of the three proposed pathways. Heterogeneity analyses reveal that content centered on digital economics positively affects health; intensive internet usage adversely impacts psychological health; individuals aged above 60 benefit in both physical and psychological health, whereas those below 60 see improvements only in psychological health. Concerning health insurance, those insured experience health gains with internet use, while the uninsured do not see notable physical health enhancements even with internet access. Regions with higher digital economic levels witness greater health benefits from the internet compared to less digitally advanced areas. Based on these findings, the paper proposes recommendations to optimize the beneficial health impact of the internet on people.

Firstly, medical service providers should proactively establish online platforms and integrate informational resources to address the public’s internet information needs, ensuring accuracy in the process. Secondly, governmental bodies must bolster internet infrastructure development, enhancing the regional digital economy to meet people’s requirements for internet facilities and hardware. Strengthening the oversight of online content is paramount; prompt removal and punitive measures should target misinformation. Concurrently, the promotion of health insurance policies is vital to alleviate individuals’ medical expenses, thereby maximizing the internet’s positive health effects. Thirdly, individuals should maintain a balanced internet usage intensity to avoid potential psychological issues arising from over-reliance. Embracing the internet’s convenience in healthcare and daily life is encouraged. Younger individuals should assist the elderly in navigating the internet, bridging the digital divide across age groups. For the elderly, prioritize using posters and printed materials in public places like community centers, courtyards, and parks where they frequently visit. Additionally, use TV, newspapers, and magazines for promotion. Secondly, consider digital platforms such as WeChat, QQ, Weibo, and official accounts of medical institutions. Use care mode features like larger fonts and buttons, and offer video calls with doctors instead of text descriptions to simplify the process. Implement facial recognition for logging into personal accounts, eliminating the need for complex password entry.
